# Second Trimester Interstitial Ectopic Pregnancy

**DOI:** 10.3390/reports8040229

**Published:** 2025-11-10

**Authors:** Daniel Reyes, Amanda Key, Zachary LeBaron, Samantha Matz, Daniel Gridley

**Affiliations:** 1 College of Medicine-Phoenix, University of Arizona, Phoenix, AZ 85004, USA; 2 Department of Radiology, Creighton University School of Medicine, Phoenix, AZ 85012, USA; 3 Department of Radiology, Valleywise Health Medical Center, Phoenix, AZ 85008, USA

**Keywords:** ectopic, interstitial, pregnancy, fallopian tube

## Abstract

**Background and Clinical Significance**: Interstitial ectopic pregnancy is a rare but potentially life-threatening type of ectopic gestation that carries high risk of rupture and hemorrhage due to its vascular location and predisposition for delayed presentation. **Case Presentation**: We present a 33-year-old female with acute abdominal pain and elevated β-hCG, with transvaginal ultrasound demonstrating a live left adnexa ectopic pregnancy dated at approximately 14 weeks. MRI confirmed the gestational sac as tubal ectopic, but poorly localized within the interstitial fallopian tube. Exploratory laparoscopy revealed the gestational sac mainly in the interstitial left fallopian tube without rupture or distal involvement. **Conclusions**: This case demonstrates absent classic imaging findings associated with tubal, interstitial, and ovarian ectopic gestations including tubal ring sign, interstitial line sign, endo-myometrial mantle measurement, or claw sign due to location and advanced gestational age. Familiarity with these challenges and recognition that interstitial ectopic pregnancy may present atypically are important for timely recognition and management.

## 1. Introduction and Clinical Significance

Interstitial ectopic pregnancy (IEP) describes a pregnancy implanted into the proximal, interstitial segment of the fallopian tube, and makes up approximately 2–4% of ectopic pregnancies [1]. Interstitial pregnancies differ from other tubal locations in that the gestational sac implants within the myometrium, often resulting in a delayed diagnosis and increased risk of catastrophic hemorrhage if rupture occurs [2]. Rarely, an ectopic pregnancy can progress to the second trimester. As the gestation grows larger in size, imaging features characteristic of specific implantation sites within the adnexa become ambiguous. While certainty of localization may prove difficult, this is an important radiologic entity to understand given its high association with maternal mortality.

## 2. Case Presentation

**Figure 1:**
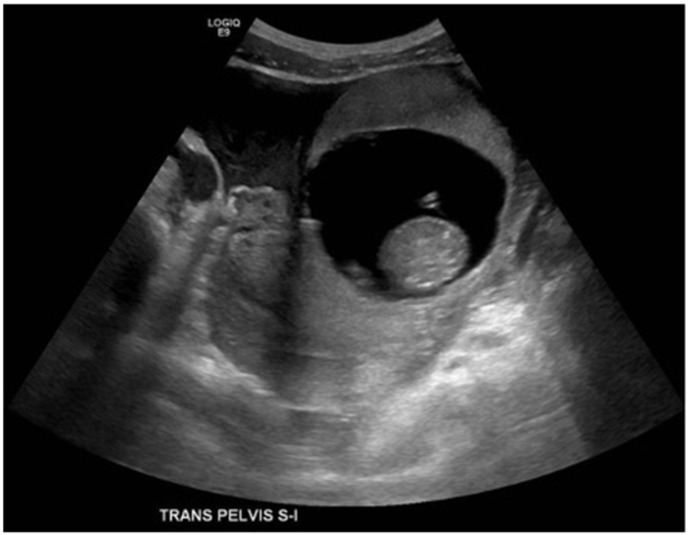
A 33-year-old female presented with diffuse abdominal pain. Transverse ultrasound of the pelvis through the level of the uterus demonstrating gestational sac outside of the uterus and free pelvic fluid. Estimated live extrauterine gestational age of 13 weeks 6 days +/− 1 week 2 days. It is unclear whether the ectopic pregnancy is located within the left fallopian tube or involves the left ovary.

**Figure 2:**
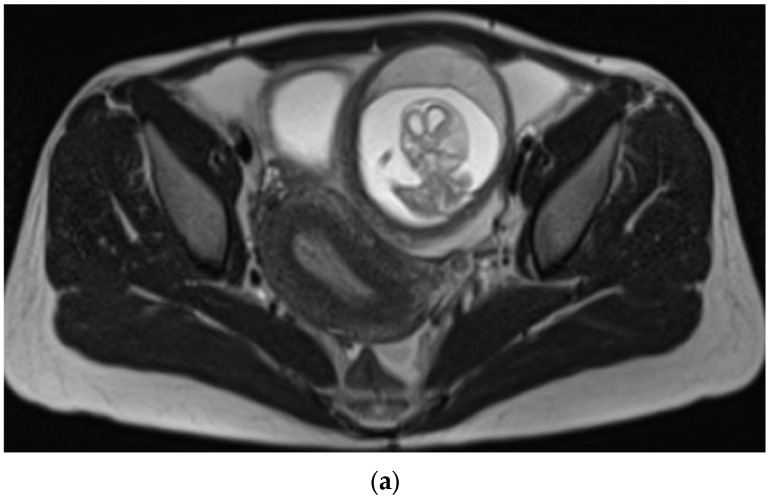

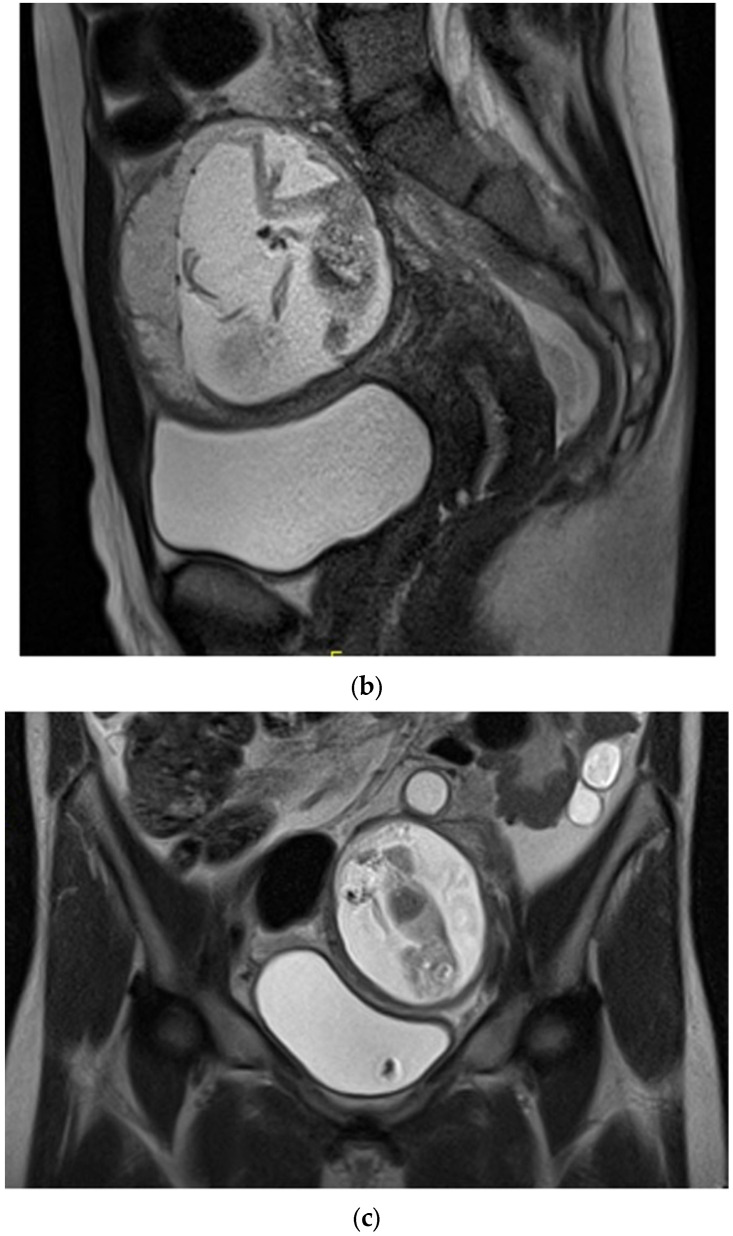
MRI without contrast showed that the ectopic gestational sac exerted mass effect upon the uterus and bladder, and was associated with a moderate amount of pelvic fluid. (**a**) Axial T2 HASTE demonstrating ectopic pregnancy in the left adnexa with associated mass effect on the bladder and uterus which deviate to the right. The placenta is located anteriorly within the gestational sac. (**b**) Sagittal T2 HASTE demonstrating ectopic pregnancy located anterior to uterine fundus and superior to bladder. The anterior wall of the gestational sac is located approximately 1.3 cm deep to the overlying skin surface. There is a moderate amount of complex free pelvic fluid. (**c**) Coronal T2 HASTE demonstrating intact gestational sac superior to the bladder in the left adnexa. The left ovary is partially visualized superior to the gestational sac and contains an incidental 2.0 cm follicle.

**Figure 3:**
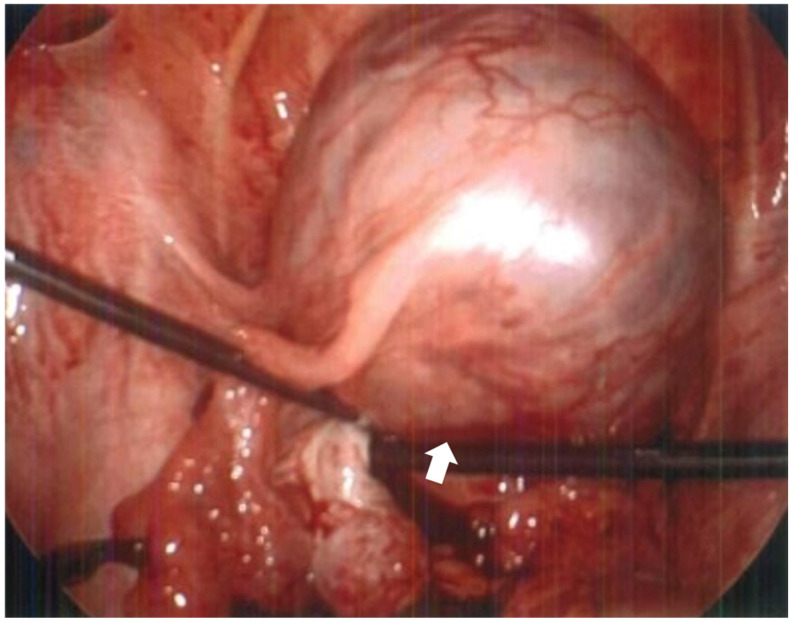
Intraoperative photo taken during diagnostic laparoscopy demonstrating intact gestational sac within dilated portion of left fallopian tube. The gestational sac (arrow) is located primarily within the interstitial portion of the fallopian tube with no involvement of the distal end. There is increased musculature surrounding the fallopian tube which may represent extended myometrium from the uterine cornua.

A 33-year-old G0P0 female presented to the emergency department with sudden, severe, and diffuse abdominal pain most prominent in the left lower quadrant, radiating to her lower back, and partially alleviated by changes in position. She had mild cramps the prior evening and a history of irregular menses with her last menstrual period occurring six months prior, and denied systemic symptoms including fever, chills, or vaginal or urethral discharge. She was stable on examination but had deep tenderness in the lower quadrants with guarding but no rebound. Urine pregnancy test was positive, and β-HCG measured 47,694 mIU/mL. Ultrasound (Figure 1) revealed a live extrauterine pregnancy in the left adnexa, corresponding to a gestational age of 13 weeks and 6 days. On MRI without contrast (Figure 2), the ectopic gestational sac was poorly localized in the fallopian tube, exerting mass effect upon the uterus and bladder. Exploratory surgery revealed the gestational sac mainly in the interstitial portion of the left fallopian tube without rupture or distal involvement, and the patient underwent salpingectomy without complications. The shape of the uterus and endometrial cavity, as well as the contralateral tube and ovary, appeared normal intraoperatively aside from the mass effect by the gestational sac, also consistent with all of the available imaging views of these structures. The patient remained hemodynamically stable and postoperative course was uncomplicated. Pathology confirmed fetal material, fragments of fallopian tube, immature chorionic villi, and benign smooth muscle favored to be myometrium (Figure 3).

## 3. Discussion

Interstitial ectopic pregnancy (IEP) refers to a pregnancy in which the blastocyst implants within the interstitial (proximal) segment of the fallopian tube, which penetrates the myometrium. Due to the expansile ability of the myometrium in this location, interstitial ectopic pregnancies can grow to advanced gestational age compared to ectopic implantations in more distal aspects of the fallopian tube [2,3,4,5]. Diagnostic delay is common as a result, potentially as late as the second trimester, and rupture may occur in over 15% of cases [2,4]. The interstitial segment of the fallopian tube is highly vascularized, increasing the risk of severe hemorrhage if rupture occurs [2]. Upon rupture, the proximity to major uterine and ovarian vessels can cause rapid, life-threatening hemoperitoneum, contributing to a maternal mortality rate that is nearly sevenfold higher than that of other ectopic pregnancy locations [1,2,3,6]. Cases which are diagnosed before rupture most commonly present as abdominal pain and vaginal bleeding, but symptoms may vary and are often vague [2,3]. Risk factors for interstitial implantation include prior salpingectomy, previous ectopic pregnancy, pelvic inflammatory disease, and conception using assisted reproductive technologies, though it may also occur without identifiable risk factors [3]. Such pregnancies represent only 2–4% of all ectopic gestations [3], but are important for radiologists to recognize given their association with high maternal morbidity and mortality.

Imaging is necessary for diagnosis, with transvaginal ultrasound performed as the preferred initial imaging study and typically demonstrates a gestational sac that is eccentrically located at the superior uterine fundus. In cases of questionable eccentricity, an endo-myometrial mantle measurement can be useful to diagnose interstitial implantation and can demonstrate separation from the uterine cavity by a thin, <5 mm, asymmetric mantle of myometrial tissue that extends laterally to encircle the gestational sac [3,4,5]. Ultrasound may also demonstrate an echogenic line, referred to as the “interstitial line sign,” which extends from the endometrial canal to the uterine cornu adjacent to the margins of the intramural gestational sac, and is thought to represent the interstitial portion of the tube or endometrium [3,4]. Three-dimensional ultrasound techniques, particularly with color Doppler, may better delineate the relationship of the gestational sac to the uterine wall and myometrium and demonstrate the interstitial location of the pregnancy [6,7]. In addition, transvaginal 2D Doppler ultrasound may reveal findings such as intense, peri-trophoblastic vascularity with numerous tortuous vessels forming a prominent vascular ring around the gestational sac [6,7].

MRI may be useful in non-urgent cases for surgical planning when diagnosis is equivocal or cases of abnormal uterine anatomy due to the ability to visualize the entire uterus and to identify the site of implantation, the relation of the gestational sac to myometrial tissue, and any associated hemoperitoneum or myometrial defect [3,7,8]. An uninterrupted junctional zone separating the endometrium from the gestational sac on MRI can confirm the diagnosis [8]. The condition can also be seen on CT, most often as an incidental discovery, which may show a ring-enhancing mass in the area of the uterine horn adjacent to the uterine fundus, and potentially myometrium partially surrounding the gestational sac [8].

Our case presented difficulty in interstitial localization on both ultrasound and MRI imaging modalities, with the advanced gestational age and size being the chief contributing factor to this ambiguity. The gestational sac appeared extrauterine on imaging, negating the usefulness of an endo-myometrial mantle measurement on ultrasound or uninterrupted junctional zone finding on MRI. An “interstitial line sign” extending from the endometrial canal was also absent on ultrasound. The substantial size of the gestational sac and the lack of “claw sign,” or partial envelopment from the primary structure, suggested the ectopic pregnancy was not ovarian in origin, however its specific implantation site could not be definitely determined on imaging.

Early presentations of IEP may be mistaken for intrauterine pregnancy, but more often possess imaging hallmarks helpful in distinguishing between the two entities. Late presentations of IEP may be more obviously extrauterine but lose the imaging characteristics indicating its interstitial source. Radiologists should be aware of IEP as a differential diagnosis for late ectopic gestations without conclusive origin and work within a multidisciplinary team for efficient patient management.

Management options depend on hemodynamic stability, gestational size, and whether rupture has occurred [1,2,3,9,10]. Early, unruptured cases may be treated using systemic or local methotrexate (success rates up to 90% under optimal criteria), with priority given to surgical management (cornual wedge resection, cornuostomy, or salpingectomy) if medical treatment fails or if rupture occurs [10]. Conservative, fertility-sparing approaches are possible in select cases, but standard surgical intervention remains crucial for emergencies [10]. The prognosis depends heavily on the timing of diagnosis and intervention; intervention before rupture reduces morbidity, and follow-up is indicated as there is risk of recurrence subsequent pregnancies [2,9].

## 4. Conclusions

IEP is a rare radiologic diagnosis associated with high maternal mortality. Delay in presentation contributes to poor diagnostic localization and increased maternal risk. Familiarity with its presentation and imaging features improves timely recognition and optimizes outcomes.

## Data Availability

The original data presented in the study are included in the article as applicable; further inquiries can be directed to the corresponding author.

## Abbreviations

The following abbreviations are used in this manuscript:

IEP	Interstitial Ectopic Pregnancy
MRI	Magnetic Resonance Imaging
CT	Computed Tomography
